# The Role of Stigma and Resilience in Healthcare Engagement Among Transgender Latinas in the U.S. South: Baseline Findings from the *ChiCAS* Study

**DOI:** 10.1007/s10903-024-01605-6

**Published:** 2024-05-29

**Authors:** Tamar Goldenberg, Amanda E. Tanner, Tucker McGuire, Jorge Alonzo, Lilli Mann-Jackson, Lucero Refugio Aviles, Carla A. Galindo, Patricia A. Bessler, Cari Courtenay-Quirk, Manuel Garcia, Beth A. Reboussin, Scott D. Rhodes

**Affiliations:** 1 Department of Public Health Education, University of North Carolina Greensboro, 1408 Walker Avenue 437 Mary Channing Coleman Building Greensboro, Winston-Salem, NC 27402 USA; 2 Department of Social Sciences and Health Policy, Division of Public Health Sciences, Wake Forest University School of Medicine, Winston-Salem, USA; 3 Division of HIV Prevention, Centers for Disease Control and Prevention, Atlanta, GA USA; 4 Department of Biostatistics and Data Science, Division of Public Health Sciences, Wake Forest University School of Medicine, Winston-Salem, NC USA

**Keywords:** Transgender women, Latine, Stigma, Healthcare

## Abstract

Research demonstrates that stigma and resilience influence transgender peoples’ healthcare use. Less is known about transgender Latinas in the U.S. South who face multilevel barriers to healthcare access. We used baseline data from the *ChiCAS* intervention study. Using logistic regression, we examined how stigma (*perceived discrimination* related to gender identity, race/ethnicity, sexual behavior and perceived documentation status and *internalized transphobia*), and resilience (*ethnic group pride* and *social support*) are associated with two healthcare outcomes (use of routine medical care and medically supervised gender-affirming hormones). We also explored barriers to accessing both types of care. After removing 13 participants with missing data, our sample size was 131 transgender Latinas in the U.S. South. Most participants (74.8%, *n* = 98) received routine medical care in the past year and 57.3% (*n* = 75) had ever received medically supervised gender-affirming hormones. Reports of discrimination were highest for gender identity and documentation status. Race/ethnicity-based discrimination was positively associated with accessing routine medical care in the past year (OR = 1.94, *p* = 0.048). Having more social support was positively associated with care (routine care: OR = 3.48, *p* = 0.002 and gender-affirming hormones: OR = 2.33, *p* = 0.003). The most commonly reported barriers to accessing both types of care included cost, insurance, and not knowing where to go. Findings highlight the importance of social support for healthcare use among transgender Latinas. Social support may be especially important when considering the unique experiences of discrimination faced by transgender Latinas in the U.S. South.

## Introduction

In the United States, transgender and other gender diverse (trans) people (including non-binary, gender queer, etc.) encounter pervasive stigma and discrimination which limits access to housing, employment, social services, and healthcare [1–6] and contributes to health inequities across multiple health outcomes, including HIV, violence, substance use disorders, mental health, and suicide [6–12]. Stigma and discrimination can be particularly challenging for trans Latinas accessing health care, given their multiple intersecting identities (e.g., gender, race/ethnicity, and documentation status) and barriers to healthcare access, such as language and transportation [13–15]. However, limited research has explored the role of stigma and resilience on healthcare use among trans Latinas in the U.S. South.

In the 2015 U.S. Trans Survey [8], with nearly 28,000 trans people in the United States (including 1,473 Latine^1^ trans people), Latine respondents reported higher rates of unemployment, poverty, and unstable housing than participants overall. In addition, 32% of Latine participants who had seen a healthcare provider in the past year reported having a negative experience related to their trans identity [8]. For trans people in general (including trans Latinas), it is important to understand experiences accessing both routine and gender-affirming medical care. For trans people who want to use it, gender-affirming medical care has been found to be associated with improved mental and physical health outcomes and improved quality of life [13, 16, 17]. However, experiences of stigma related to multiple aspects of identity may limit access to both types of care [18].

### Theoretical Considerations

Minority stress theory [19–21] is useful for understanding how stigma influences healthcare use among trans Latinas through a focus on distal (e.g., discrimination and victimization) and proximal (e.g., internalized and anticipated stigma) minority stressors as well as resilience (i.e., strategies for responding to stress exposure). Minority Stress Theory recognizes that anti-trans stigma can contribute to poorer mental and physical health, including access to and use of health care [19–25]; this occurs when trans people anticipate or experience mistreatment within healthcare settings, which results in receiving poorer care and healthcare avoidance [8, 13, 26, 27]. Research has also demonstrated that experiencing anti-trans stigma outside of the healthcare context can reduce use of healthcare services [22].

Minority Stress Theory posits that resilience strategies for responding to stress exposure may ameliorate the negative health consequences caused by minority stress [28, 29]. Resilience factors occurring within healthcare settings (e.g., providers who offer gender-affirming support) and outside of healthcare settings (e.g., ethnic group pride, social support, and community connectedness) may help to improve use of healthcare services for trans people [13, 18, 22, 30].

While prior research explored the role of stigma and resilience on healthcare use [18, 22, 25, 26, 28], it is unclear how the intersecting forms of stigma experienced by trans Latinas (e.g., related to gender identity, race/ethnicity, and perceived documentation status) may influence use of health care, including general health care experiences and access to medically supervised gender-affirming care. When considering the experiences of trans Latinas, it is important to consider an intersectionality framework [31–34], which identifies that multiple systems of oppression (e.g., racism, sexism, and cisgenderism) occur simultaneously and reinforce each other in ways that change the experiences of people with multiple marginalized identities. As such, trans Latinas’ experiences with multiple and intersecting forms of stigma and discrimination may amplify consequences for healthcare utilization. These experiences may be exacerbated in the U.S. South, where policies are simultaneously targeting trans and Latine communities, limiting access to health care (including medically supervised gender-affirming care), and potentially increasing experiences of stigma inside and outside of healthcare settings [6, 35–38]. Accordingly, this analysis examines associations between multiple forms of stigma, resilience, and healthcare use among trans Latinas living in the U.S. South.

## Methods

### Study Design

This analysis uses baseline data from a two-group, randomized, intervention-waitlist control study designed to evaluate the efficacy of the *ChiCAS: Chicas Creando Acceso a la Salud* [*ChiCAS: Girls Creating Access to Health*] intervention. Briefly, *ChiCAS* is a small group, two-session intervention (delivered in person or virtually [39]) designed to increase PrEP uptake, consistent condom use, and use of medically supervised gender-affirming hormone therapy among Spanish-speaking HIV seronegative trans Latinas who have sex with men (see [39–41] for more details).

From July 2019 to July 2021, trans Latinas were recruited across North and South Carolina by distributing study information in various locations (e.g., clubs, LGBTQ + organizations, community colleges, Hispanic/Latine-owned businesses, and social media) and through word of mouth. Eligibility criteria included: (1) self-identifying as a trans woman or having been assigned male sex at birth and self-identifying as female, (2) self-identifying as Hispanic/Latina, (3) being 18 years of age or older, (4) reporting sex with at least one man in the past 6 months, (5) being HIV negative (self-report verified by rapid HIV testing), (6) speaking fluent Spanish, and (7) providing informed consent. Persons who had participated in any HIV prevention interventions in the past 12 months were ineligible.

We recruited and enrolled 144 trans Latinas (162 people were screened in total; 1 person did not meet eligibility criteria and 16 were unable to participate in the study intervention). Participants completed an interviewer administered REDCap baseline assessment. The Institutional Review Board of Wake Forest University School of Medicine provided human subject oversight.

### Measures

Dependent variables included healthcare outcomes measures and independent variables included measures of stigma, resilience, and covariates.

#### Healthcare Outcomes

We measured two binary outcomes examining whether participants (1) received routine medical care in the United States in the past year and (2) ever received gender-affirming hormones from a medical provider in the United States.

#### Stigma

Stigma was measured through two components – *perceived discrimination* and *internalized transphobia*. *Perceived discrimination* was measured using a modified version of the Everyday Discrimination scale [42], previously used with trans Latinas in North Carolina [43]. We examined four types of discrimination – related to race/ethnicity, transgender identity, sexual behavior, and others’ perceptions of the participant’s documentation status. For each type, participants were asked three questions based on experiences with unfair treatment, violence, and discrimination. Participants indicated their agreement to these questions on a 4-point scale (1 = strongly disagree-4 = strongly agree). Mean scores were calculated with higher scores indicating more agreement that the participant often experiences discrimination. Discrimination measures had high internal reliability (Cronbach’s alphas: race/ethnicity = 0.91, transgender identity = 0.89, having sex with men = 0.92, perceptions of documentation status = 0.90).

We also measured *internalized transphobia* using the Internalized Transphobia Scale [44]. This 26-item measure used the mean scores from participants’ level of agreement (1 = strongly disagree-7 = strongly agree) on questions about experiences with pride, passing, alienation, and shame related to being transgender (Cronbach’s alpha = 0.78).

#### Resilience

We included two resilience measures: *ethnic group pride* and *social support* as both are associated with improved health and healthcare outcomes among trans populations [22, 24]. *Ethnic group pride* was measured using the Multigroup Ethnic Identity Measure [45] taking the mean score of 12 items asking participants’ agreement (1 = strongly disagree-4 = strongly agree) with statements about experiences with and pride in their ethnicity. This scale has been used with Hispanic/Latine populations [45] and had good internal reliability (Cronbach’s alpha = 0.84).

*Social support* was measured using an adapted version of the Index of Sojourner Social Support (ISSS) Scale [46]. Participants were asked, on a scale of 0 (no one would do this) to 4 (most would do this), to indicate if they knew anyone who would perform a series of 18 actions (e.g., “Comfort you when you feel homesick”). Mean scores were calculated with higher scores indicating more social support. This scale has been previously used with Hispanic/Latine populations [46, 47] and had good internal reliability (Cronbach’s alpha = 0.98).

#### Healthcare Barriers

Participants were asked to identify specific reasons that had prevented them from seeking health care in the United States in the past 12 months, with separate questions for general care and medically supervised hormone therapy. Participants selected all that applied from a list of 13 barriers (e.g., no transportation, thought the medical bill would be too high). Barriers were identified based on data previously collected by our research team [15, 48, 49] and were adapted to apply to general health care and medically supervised hormone therapy.

#### Demographics

We collected information on age, education, employment, time in the United States, and country of origin. Age was measured as a continuous variable in years. Education was measured as a binary variable based on receipt of a high school diploma or GED, based on prior research and the distribution of education among this sample [14, 50]. Employment was measured as a binary variable based on year-round employment. Time in the United States was measured as a continuous variable in years. These demographic variables were selected because they have been found to be associated with routine medical care and use of medically supervised gender-affirming hormones among transgender people in the United States [36, 37]. A focus on time in the United States is also important since the healthcare outcome questions specifically ask about care that has been received in the United States, providing some participants with more opportunity to receive care.

#### Analysis

Data were analyzed using Stata 14 (College Station, Texas). None of the independent variables demonstrated multicollinearity. To reduce missing data, for the internalized transphobia, ethnic group pride, and social support scales, mean scores were calculated for participants who answered at least 80% of items. After adjusting calculations for these scales based on missing data, fewer than 10% of participants (*n* = 13) had missing data on any variable included in the analysis and data were missing at random (no differences between participants missing vs. not missing data on outcomes or demographic variables), so participants with missing data were excluded from analyses, resulting in a sample of 131 participants.

Descriptive statistics assessed the sample distributions of all variables. We fit unadjusted and adjusted logistic regression models to examine associations between stigma (discrimination based on race/ethnicity, transgender identity, sexual behavior, perceptions of documentation status, and internalized transphobia), resilience (ethnic group pride and social support), and the healthcare outcomes (receiving routine care in the past year and ever receiving medical care for hormones). Multivariable logistic regression models also controlled for demographic variables.

To better understand the associations between stigma, resilience, and healthcare outcomes, in the context of other barriers to accessing care, we also examined the frequency of specific barrier responses.

## Results

Participants had a mean age of 33.1 (SD = 9.61; range 18–59), most were born in Mexico (65.7%, *n* = 86), and time living in the United States ranged from 0 to 37 years (Mean = 16.0, SD = 8.63). About half of the participants had received a high school diploma or GED (48.1%, *n* = 63) and approximately two-thirds (62.6%, *n* = 82) were employed year-round. See Table 1.

**Table 1:** Descriptive statistics (*n* = 131)

Variable	% (*n*)	Mean (SD)	Observed Range
Healthcare use variables			
Received routine medical care in U.S. within past year	74.81 (98)		
Ever received gender-affirming hormones in the U.S. from a medical provider	57.25 (75)		
Stigma variables			
Perceived discrimination based on race/ethnicity^a^		1.96 (0.94)	1.00–4.00
Perceived discrimination based on transgender identity^a^		2.53 (1.06)	1.00–4.00
Perceived discrimination based on having sex with men^a^		1.50 (0.65)	1.00–4.00
Perceived discrimination based on documentation status^a^		2.06 (1.02)	1.00–4.00
Internalized transphobia^b^		2.50 (0.78)	1.00-4.88
Resilience variables			
Ethnic group pride^c^		3.08 (0.51)	1.67-4.00
Social support^d^		1.67 (0.86)	0.56-4.00
Demographic variables			
Age		33.13 (9.61)	18–59
Number of years living in U.S.		15.99 (8.63)	0–37
Received high school diploma or GED	48.09 (63)		
Employed year-round	62.60 (82)		
Country/region of birth			
United States	11.45 (15)		
Mexico	65.65 (86)		
Central America (Honduras, El Salvador, and Guatemala)	21.37 (28)		
South America (Argentina)	0.76 (1)		
Caribbean (Cuba)	0.76 (1)		

^a^ Total possible range for all perceived discrimination scales is 1–4

^b^ Total possible range for the internalized transphobia scale is 1–7

^c^ Total possible range for the ethnic group pride scale is 1–4

^d^ Total possible range for the social support scale is 0–4

Three-quarters of participants (74.8%, *n* = 98) had received routine medical care in the United States in the past year and 57.3% (*n* = 75) had ever received gender-affirming hormones from a medical provider in the United States. Participants reported high levels of discrimination, especially related to their transgender identity and perceptions related to their documentation status, with means above 2 for each of these scales (scale 1–4, where 4 means that participants strongly agree with statements related to experiences of discrimination). Reports of internalized anti-trans stigma were not as high, with a mean of 2.50 (SD = 0.78; scale 1–7, with higher numbers indicating more agreement with experiences of internalized stigma). Participants scored high on ethnic group pride, with a mean of 3.08, (range 1–4, with 4 indicating more pride). Participants had lower scores for social support, with a mean score of 1.67 on the social support scale (range of 0–4, with 4 indicating more social support).

When examining bivariable analyses with unadjusted logistic regression, social support and ethnic group pride were statistically associated with the two healthcare outcomes (Table 2). Having more social support was positively associated with getting routine medical care in the United States in the past year (unadjusted OR = 2.76, *p* = 0.002) and positively associated with ever getting gender-affirming hormones from a medical provider in the United States (unadjusted OR = 2.47, *p* < 0.001). Participants who scored higher on the ethnic group pride scale were also more likely to have accessed gender-affirming care from a medical provider in the United States (unadjusted OR = 2.57, *p* = 0.012). No other stigma variables were significantly associated with the health outcomes. For the demographic variables, graduating high school was positively associated with both healthcare use outcomes (unadjusted OR for routine medical care = 2.71, *p* = 0.020; unadjusted OR for gender-affirming hormone use = 2.76, *p* = 0.006) and being employed year-round was negatively associated with accessing gender-affirming hormones from a medical provider in the United States (unadjusted OR = 0.38, *p* = 0.012).

**Table 2:** Bivariate unadjusted logistic regression models examining healthcare outcomes (*n* = 131)

	Received routine medical care in U.S. within past year			Ever received gender-affirming hormones from a medical provider in the U.S.		
	Unadjusted OR	95% CI	p-value	Unadjusted OR	95% CI	p-value
Stigma variables						
Perceived discrimination based on race/ethnicity	1.30	0.83, 2.01	0.248	1.00	0.69, 1.44	0.979
Perceived discrimination based on transgender identity	0.70	0.47, 1.04	0.078	1.04	0.75, 1.44	0.834
Perceived discrimination based on having sex with men	1.04	0.56, 1.91	0.908	0.98	0.58, 1.68	0.954
Perceived discrimination based on documentation status	0.83	0.57, 1.22	0.343	0.93	0.66, 1.30	0.661
Internalized transphobia	1.09	0.65, 1.83	0.744	1.30	0.82, 2.06	0.259
Resilience variables						
Ethnic group pride	1.27	0.58, 2.80	0.548	2.57	1.23, 5.37	0.012*
Social support	2.76	1.47, 5.19	0.002*	2.47	1.50, 4.07	< 0.001*
Demographic variables						
Age	0.99	0.95, 1.04	0.789	0.98	0.95, 1.02	0.280
Number of years living in U.S.	0.98	0.94, 1.03	0.420	0.98	0.94, 1.02	0.391
Received high school diploma or GED	2.71	1.17, 6.29	0.020*	2.76	1.35, 5.67	0.006*
Employed year-round	0.94	0.42, 2.14	0.886	0.38	0.18, 0.81	0.012*

*Statistical significance at *p* < 0.05

Table 3 summarizes multivariable logistic regression model results. Experiencing more discrimination based on race/ethnicity was associated with being more likely to get routine care (OR = 1.94, *p* = 0.048). Social support was positively associated with both routine care (OR = 3.48, *p* = 0.002) and gender-affirming care from a medical provider in the United States (OR = 2.33, *p* = 0.003). Educational attainment was also significantly associated with both forms of health care; participants who graduated from high school/had a GED had 3.23 times the odds of getting routine medical care in the past year (*p* = 0.022) and 3.14 times the odds of ever using gender-affirming hormones from a medical provider (*p* = 0.011), compared with those who did not graduate high school. In addition, being employed year-round (OR = 0.31, *p* = 0.010) was negatively associated with using gender-affirming hormones from a medical provider.

**Table 3:** Multivariable logistic regression models examining healthcare outcomes (*n* = 131)

	Received routine medical care in U.S. within past year			Ever received hormones from a medical provider in the U.S.		
	aOR^a^	95% CI	p-value	aOR^a^	95% CI	p-value
Stigma variables						
Perceived discrimination based on race/ethnicity	1.94	1.01, 3.73	0.048*	0.88	0.50, 1.53	0.640
Perceived discrimination based on transgender identity	0.69	0.41, 1.16	0.163	1.10	0.70, 1.74	0.673
Perceived discrimination based on having sex with men	0.82	0.36, 1.86	0.642	1.07	0.50, 2.28	0.858
Perceived discrimination based on documentation status	0.54	0.29, 1.03	0.060	0.64	0.36, 1.14	0.133
Internalized transphobia	1.06	0.55, 2.04	0.870	1.60	0.86, 2.97	0.137
Resilience variables						
Ethnic group pride	0.66	0.22, 1.97	0.453	2.02	0.76, 5.34	0.159
Social support	3.48	1.61, 7.54	0.002*	2.33	1.34, 4.06	0.003*
Demographic variables						
Age	1.05	0.98, 1.12	0.152	1.03	0.97, 1.09	0.298
Number of years living in U.S.	0.95	0.89, 1.02	0.158	0.98	0.93, 1.04	0.547
Received high school diploma or GED	3.23	1.19, 9.29	0.022*	3.14	1.31, 7.56	0.011*
Employed year-round	0.84	0.31, 2.26	0.723	0.31	0.13, 0.76	0.010*

*Statistical significance at *p* < 0.05

^a^All variables in the table were included for adjustment in the multivariable logistic regression models

Participants reported similar reasons for not accessing routine medical care and gender-affirming hormones (Table 4). The most common reasons were concerns related to high healthcare costs, not having health insurance, and not knowing where to go to receive services.

**Table 4:** Other barriers to accessing health care (*n* = 131)

	Reasons for not seeking routine medical care in U.S. within past year, % (*n*)	Reasons for not seeking hormones in the U.S. within the past year, % (*n*)
Did not have health insurance	20.77 (27)^a^	24.43 (32)
The clinic, health department, or hospital was too far away	8.46 (11)^a^	12.98 (17)
Did not have transportation	10.69 (14)	9.16 (12)
Could not take time off from work	5.38 (7)^a^	9.92 (13)
The clinic, health department, or hospital was not open when participant could go	6.87 (9)	7.63 (10)
The staff and providers did not speak participant’s language	4.58 (6)	4.58 (6)
Not sure where to go for the services needed	16.03 (21)	26.72 (35)
Took too long to get an appointment	6.87 (9)	9.16 (12)
During previous visits, it took too long to see a doctor	4.62 (6)^a^	6.92 (9)^a^
Felt that would be treated poorly	12.21 (16)	12.98 (17)
Did not know whether eligible to be seen	12.98 (17)	19.85 (26)
Concerned about other people finding out about participant’s health	4.58 (6)	9.16 (12)
Thought medical bill would be too high	28.46 (37)^a^	27.69 (36)^a^

^a^*n*=130 due to missing data

## Discussion

Overall, nearly three-quarters of participants accessed routine medical care in the United States in the past year and more than half accessed gender-affirming hormones from a medical provider in the United States in their lifetime. Considering the common negative experiences that trans people have with health care [18, 51], participants reported relatively high levels of access to routine medical care, and reports of medically supervised gender-affirming hormones were consistent with national findings among trans populations [8]. However, reports of stigma related to transgender identity and perceptions of documentation status were high and participants identified many barriers to accessing routine care, with 15–25% of participants reporting concerns about healthcare costs, health insurance, and knowledge about where to receive services. Similarly, in the U.S. Trans Survey, Latine respondents reported not seeing a provider in the past year due to cost (37%) and due to negative experiences within healthcare settings (26%) [8]. Minority Stress Theory [19–21] and previous empirical findings [18, 22, 23, 28] identify that both minority stressors and resilience factors are important for healthcare access; our results extend these findings to demonstrate these associations for trans Latinas in the U.S. South, while also highlighting additional barriers for accessing care.

Discrimination based on race or ethnicity was positively associated with accessing routine medical care. These findings are not as expected, since race-based discrimination is typically associated with reduced access to resources, including health care [15]. However, since findings are cross-sectional, it is possible that participants who accessed health care may have experienced race/ethnicity-based discrimination within healthcare settings [52, 53]. It is also possible that participants who accessed health care may have been more likely to also engage in other systems that perpetuate more race/ethnicity-based discrimination.

Results highlight that social support is an especially important factor for trans Latinas (associated with both healthcare use outcomes in both unadjusted and adjusted models). Having more social support was associated with increased odds of accessing both routine and gender-affirming care. Understanding social support as a resilience factor that may increase healthcare use is important in intervention development for trans Latinas. Since reports of social support were low among the participants, innovative interventions for improving social support may be needed. Additionally, although not significant in the multivariable models, in the bivariate models, ethnic group pride (the other resilience factor) was associated with increased use of gender-affirming hormones. Taken together, these findings highlight the importance of considering strengths-based approaches for resisting stigma and improving access to health care among trans Latinas.

Some demographic variables were associated with the healthcare outcomes. Graduating high school/receiving a GED was positively associated with both healthcare outcomes, while being employed year-round was negatively associated with gender-affirming care. Findings related to education are consistent with previous research [54]; having more education can increase access to resources, including health care. Findings on employment were more surprising; however, it is possible that participants who were employed may have been less likely to want hormones, especially if they were unable to disclose their gender identity at work [8, 28]. It is also possible that participants who were employed may have had more challenges with finding time to access care, nearly 10% of participants identified this as a barrier for accessing gender-affirming care.

### Limitations

Findings should be considered within the context of the study design. Data are cross-sectional, so causal inferences cannot be made; this is important given some of the unexpected directions of our findings. The sample size is small, but considerable, given the very specific population of trans Latinas not living with HIV in U.S. South. Still, a small sample size limits our ability to detect statistical significance. A larger sample size would allow us to explore how the resilience variables (social support and ethnic group pride) may moderate the association between the stigma variables (discrimination variables and internalized transphobia) and healthcare use outcomes. We ran post-hoc analyses to explore these interactions and did not find any statistically significant results; however, it is unclear if we were unable to detect statistical significance due to the small sample size or because no moderation occurred. A larger sample size may also allow for more complex analyses (e.g., latent class analysis) that can more fully explore the intersectionality of multiple forms of stigma [33]. Measuring and analyzing multiple forms of stigma separately can be especially challenging because participants may not be able to determine why they are experiencing discrimination (e.g., it may be hard to determine if discrimination occurred to due to race/ethnicity or trans identity).

Care should be used when generalizing results to a larger population of trans Latinas, and should be considered within the context of the eligibility criteria (e.g., Spanish fluency, not living with HIV, and sex with at least one man in the past 6 months). We did not ask about participants’ desire to use gender-affirming hormones, so participants who indicated that they did not access gender-affirming medical care included those who did not want hormones as well as those who wanted hormones but did not access them from a medical provider. Future research examining gender-affirming care should consider desire for use of gender-affirming hormones in addition to access. This analysis was limited to the variables included in the larger study, which focused on ethnic group pride, but not pride related to trans identity; future research exploring resilience and pride should consider more comprehensive measures to assess experiences with pride related to multiple identities. Finally, our models also do not include questions related to health insurance; however, we did ask if health insurance was a reason for not accessing routine care and gender-affirming hormones from a medical provider and found that this was a common barrier.

Despite limitations, this study has many strengths, including that we examined multiple forms of stigma (including perceived discrimination related to different aspects of identity and internalized transphobia), and we considered various resilience factors (social support and ethnic group pride). Furthermore, an examination of both general health care and gender-affirming hormones from a medical provider allows us to understand the nuanced experiences across these types of care.

## Conclusions

Given the burden of health issues affecting trans Latinas in the United States, it is critical to understand the factors affecting their healthcare use. This study represents a step in documenting factors that affect utilization of routine health care and medically supervised gender-affirming care. The results highlight that to improve trans Latinas’ healthcare access, interventions must consider the context of individual characteristics that may influence healthcare use (e.g., education, employment), while also focusing on interpersonal factors (e.g., social support), clinical care factors (e.g., reducing experiences of healthcare discrimination), and structural factors (e.g., reducing discrimination related to race/ethnicity and perceptions of documentation status) that may be useful mechanisms for intervention. Given the many challenges trans Latinas face, future public health research and programs should consider how to create culturally congruent interventions that increase access to care for this specific population. These interventions should consider how to reduce discrimination and improve experiences within health care and also consider strengths-based approaches that increase social support and ethnic group pride. Addressing these factors is complex but necessary to improve access, utilization, and health among this marginalized population.

## Footnotes

1 The term “Latine” uses a gender-neutral “e”, which replaces the gendered endings “a” and “o” as in “Latina” and “Latino” and is similar to “Latinx”. This term is increasingly used within Latine LGBTQ + communities.
